# Editorial: Advances in imaging of cervical cancer

**DOI:** 10.3389/fonc.2024.1421476

**Published:** 2024-06-03

**Authors:** Mingfu Wu, Alessio G. Morganti, He Du

**Affiliations:** ^1^ Department of Obstetrics and Gynecology, Tongji Hospital, Tongji Medical College, Huazhong University of Science and Technology, Wuhan, China; ^2^ Radiation Oncology, IRCCS Azienda Ospedaliero-Universitaria di Bologna, Bologna, Italy; ^3^ Radiation Oncology, DIMEC, Alma Mater Studiorum, Bologna University, Bologna, Italy; ^4^ Central Hospital of Enshi Tujia and Miao Autonomous Prefecture, Wuhan University, Enshi, China

**Keywords:** cervical cancer, imaging, molecular imaging, MRI, artificial intelligence (AI)

Central Hospital of Enshi Tujia and Miao Autonomous Prefecture, Wuhan University Enshi, China Cervical cancer (CCa) remains a significant public health challenge worldwide, with over half a million new cases diagnosed annually (1). Despite advances in screening and vaccination, it continues to be a leading cause of cancer-related deaths among women in low- and middle-income countries, where access to preventive and treatment services is often limited (2). The prognosis for advanced disease remains poor, underscoring the urgent need for enhanced diagnostic and therapeutic strategies.

In recent years, the field of oncology has witnessed remarkable advancements in diagnostic imaging technologies, significantly impacting patient care also in cervical cancer (CCa). The “*Advances in imaging of cervical cancer*” Research Topic has curated a collection of five pivotal papers that underscore recent progress in imaging techniques aimed at enhancing prediction and diagnosis in CCa. This editorial seeks to encapsulate the essence and findings of these contributions, weaving together their implications for future research and clinical practice.

Guo et al. explored the utility of synthetic magnetic resonance imaging (MRI) in evaluating lympho-vascular space invasion (LVSI) in cervical squamous cell carcinoma without lymph node metastasis. Their study underscores the potential of quantitative parameters from synthetic MRI (T1, T2, and proton density maps) in distinguishing between negative and positive LVSI groups, thereby offering a non-invasive method for assessing tumor aggressiveness. Fan et al. demonstrated the clinical value of combining conventional MRI with diffusion-weighted imaging (DWI) and the apparent diffusion coefficient (ADC) for predicting pelvic lymph node metastasis in cervical cancer. Their findings reveal that tumor ADC values are significantly lower in patients with pelvic lymph node metastasis, suggesting that this method could improve diagnostic accuracy and patient management. Zhu et al. highlighted the potential diagnostic value of superb microvascular imaging (SMI) in differentiating between premalignant and malignant cervical lesions. Their research illustrates how the vascular index on SMI can offer a high diagnostic performance, presenting a promising non-invasive option for early detection and characterization of cervical lesions. Lin et al. focused on the development of a combined model incorporating clinical characteristics and MRI features to predict survival in patients with locally advanced CCa. Their comprehensive approach indicates that integrating MRI factors with clinical characteristics can significantly enhance the predictive accuracy of prognostic models, aiding in tailored patient care. Finally, Ma et al. assessed the feasibility of using contrast-enhanced ultrasound combined with elastography for predicting the efficacy of concurrent chemoradiotherapy in CCa. Their study suggests that this combination, along with the tumor marker squamous cell carcinoma antigen, can improve the prediction of disease progression, highlighting the role of advanced imaging in therapeutic planning.

The research presented in this topic not only advances our understanding of imaging techniques in the diagnosis and staging of CCa but also opens new avenues for predicting pathological stage, lesions’ malignancy, tumor response, and clinical outcomes. Such predictive capabilities are vital for developing personalized treatment plans, potentially improving prognosis and quality of life for patients with CCa.

The integration of artificial intelligence (AI) with imaging technologies promises to further revolutionize this field, offering more precise and efficient predictive models also in CCa (3). As we venture deeper into the field of AI, other groundbreaking applications of imaging emerge, such as radiomics and the analysis of body composition. Radiomics, the extraction of a large number of features from radiographic medical images using data-characterization algorithms, offers a new dimension in understanding CCa tumor heterogeneity and could significantly impact treatment decisions and prognostication (4). Similarly, imaging-based analysis of body composition, including conditions like sarcopenia (the loss of muscle mass and strength) and sarcopenic obesity (the co-occurrence of sarcopenia and obesity), provides vital insights into patient outcomes and treatment tolerability (5). These advancements underscore the growing role of comprehensive imaging assessments in tailoring oncologic care, indicating that the future of CCa treatment lies not only in diagnosing and staging but also in a nuanced understanding of the patient’s physiological state and the tumor’s microenvironment.

As we continue to explore these technologies, our hope is that they will lead to significant improvements in the prognosis and treatment of cervical cancer, marking a new era in the management of gynecological cancers. In conclusion, the contributions within this Research Topic signify a substantial leap forward in the application of modern imaging technologies in oncology. They not only enhance our diagnostic and predictive capabilities but also pave the way for innovations that could transform patient care in cervical cancer.

## Author contributions

MW: Writing – original draft, Writing – review & editing. AM: Writing – review & editing. HD: Writing – review & editing.
